# Computer-Aided Diagnosis System for Blood Diseases Using EfficientNet-B3 Based on a Dynamic Learning Algorithm

**DOI:** 10.3390/diagnostics13030404

**Published:** 2023-01-22

**Authors:** Sameh Abd El-Ghany, Mohammed Elmogy, Abd El-Aziz

**Affiliations:** 1 Department of Information Systems, College of Computer and Information Sciences, Jouf University, Sakakah 42421, Saudi Arabia; 2 Information Technology Department, Faculty of Computers and Information, Mansoura University, Mansoura 35516, Egypt

**Keywords:** acute lymphoblastic leukemia (ALL), malaria parasite, EfficientNet-B3, learning rate (LR), deep learning, computer-aided diagnostic (CAD)

## Abstract

The immune system’s overproduction of white blood cells (WBCs) results in the most common blood cancer, leukemia. It accounts for about 25% of childhood cancers and is one of the primary causes of death worldwide. The most well-known type of leukemia found in the human bone marrow is acute lymphoblastic leukemia (ALL). It is a disease that affects the bone marrow and kills white blood cells. Better treatment and a higher likelihood of survival can be helped by early and precise cancer detection. As a result, doctors can use computer-aided diagnostic (CAD) models to detect early leukemia effectively. In this research, we proposed a classification model based on the EfficientNet-B3 convolutional neural network (CNN) model to distinguish ALL as an automated model that automatically changes the learning rate (LR). We set up a custom LR that compared the loss value and training accuracy at the beginning of each epoch. We evaluated the proposed model on the C-NMC_Leukemia dataset. The dataset was pre-processed with normalization and balancing. The proposed model was evaluated and compared with recent classifiers. The proposed model’s average precision, recall, specificity, accuracy, and Disc similarity coefficient (DSC) were 98.29%, 97.83%, 97.82%, 98.31%, and 98.05%, respectively. Moreover, the proposed model was used to examine microscopic images of the blood to identify the malaria parasite. Our proposed model’s average precision, recall, specificity, accuracy, and DSC were 97.69%, 97.68%, 97.67%, 97.68%, and 97.68%, respectively. Therefore, the evaluation of the proposed model showed that it is an unrivaled perceptive outcome with tuning as opposed to other ongoing existing models.

## 1. Introduction

The human body is supplied with essential substances by blood. The three main components of blood cells in the human body are erythrocytes (red blood cells (RBCs)), leukocytes (white blood cells (WBCs)), and thrombocytes (platelets). RBCs transport oxygen throughout the body and platelets assist with blood clotting in the event of injury. Human infections are prevented and fought by WBCs. Although WBCs only comprise one percent of the blood’s volume, even small changes can significantly impact the human immune system. Changes in the blood’s WBC count present a challenge. An abnormally high number of WBCs can prevent infection [1].

One of the most lethal diseases known is cancer, defined as abnormal and uncontrolled WBC growth [2]. The world health organization (WHO) estimates that approximately 10 million people will die from cancer in 2020, representing an increase of nearly 60% from 2000. Around 19.3 million people will be diagnosed with the disease [3]. Between now and 2040, it is anticipated that the number of people affected will increase by approximately 50% [4]. The disease’s age determines the four main types of leukemia, which are acute myelogenous leukemia (AML), chronic lymphocytic leukemia (CLL), chronic myeloid leukemia (CML), and acute lymphoblastic leukemia (ALL) [5,6]. The bone marrow, where blood cells are made, is typically where ALL, a type of blood cancer, begins. WBCs are associated with this type of cancer. The abnormal cells in intense leukemia develop and spread rapidly, requiring brief therapy, though ongoing leukemia is hard to distinguish in its beginning phases. The immune system is made more vulnerable because of the blood’s inability to perform its normal function. Additionally, most of the body is at risk due to the bone marrow’s inability to produce healthy platelets and red blood cells [7].

Bone marrow produces many abnormal WBCs in ALL patients. These WBCs have the potential to enter the bloodstream, causing harm to the liver, spleen, brain, and kidneys, among other organs, that can lead to additional cancers that could kill the human. Because ALL can disseminate throughout the body quickly, if it is not treated or diagnosed early, it can sometimes result in death. The essential information regarding ALL reveals that more than 5690 ALL cases will occur in the USA in 2021 [8]. More than 1550 people, including children and adults, are expected to die. Leukemia, and especially ALL, can be easily treated when diagnosed early. Leukemia can be challenging to diagnose because its symptoms resemble joint pain, bone pain, anemia, weakness, and fever.

In most cases, specific symptoms and signs lead doctors to suspect ALL in patients, while various clinical examinations validate the ALL diagnosis. Blood tests are frequently performed during the initial stage on suspected ALL patients. Complete blood counts and peripheral blood smears are performed to monitor changes in the number and appearance of WBCs and other blood cells. Utilizing chromosome-based tests such as cytogenetics, fluorescence in situ hybridization, and polymerase chain reaction, in which chromosomes are observed to recognize unusual blood cells, improves the accuracy of the diagnosis of ALL [4].

Hematologists normally utilize various intrusive techniques to analyze diseases. A biopsy, an invasive procedure that examines spinal fluid, bone marrow, or blood, is frequently performed [9]. During these examinations, the doctor checks to see if there are enough WBCs and other relevant physical signs to indicate the presence of ALL. These techniques are time-consuming, expensive, and painful. Because the results of these manual and expert-specific techniques are heavily dependent on the expertise and knowledge of the expert performing the analysis, they are also susceptible to error [10]. Medical image-analysis-based techniques provide quicker, safer, and less expensive solutions while avoiding the complications of such invasive procedures. It is simple to generalize image processing and computer-vision-based methods and eliminate human error.

**Figure 1:**
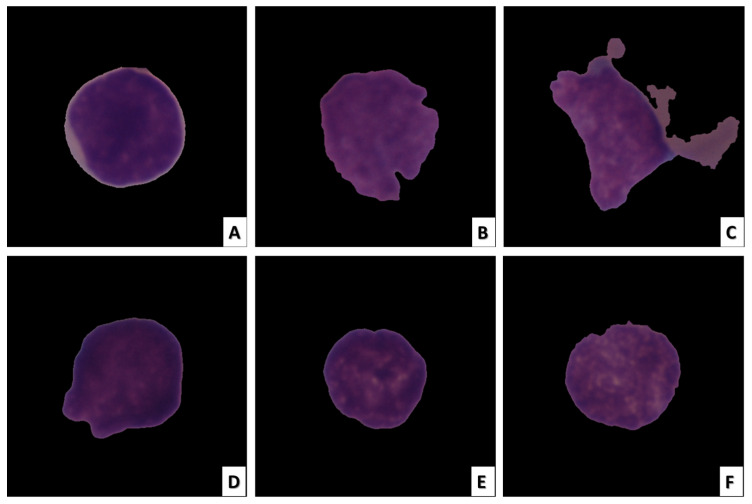
ALL cell image samples. The images in (**A**–**C**) depict ALL cancer cells, while the images in (**D**–**F**) depict healthy cells.

Radiologists can easily perform image-based analysis. Even though they are non-invasive, these techniques can still have the same drawbacks as invasive ones. When large datasets containing hundreds of thousands of medical images are required to be analyzed by human experts, the manual analysis performed by radiologists becomes extremely laborious, error-prone, and time-consuming. In medical images, particularly histopathology images, inherent texture and morphology, which are overlapping features, make the task more difficult. Figure 1 depicts images of ALL cancerous and healthy cells [11].

Because of their low interclass detachability and high intraclass homogeneity, leukocytes are difficult to distinguish. Therefore, it is essential to develop methods for the accurate and dependable identification of leukemia for prompt diagnosis and early treatment. Nonetheless, as a rule, ALL malignant growth cells vary from solid cells based on different variables, including morphology, cell size, shape, and surface [12]. A fully automated solution based on deep learning (DL) can utilize any of these attributes to recognize healthy cells and ALL disease images.

In recent years, DL, or deep neural networks (DNNs), has emerged as a cutting-edge technology for speech recognition and computer vision [13]. DL is an artificial intelligence (AI) technique considering the portrayal of information learning. DL methods employ supervised, semi-supervised, and unsupervised learning. Learning models are very different depending on the learning framework. The basis of DL is the efficient manual replacement of features using hierarchical feature extraction and semi-supervised or unsupervised feature learning [14]. Between the input layer and the output layer in DL, there are several hidden layers. It has several practical advantages. DL supports both labeled and unlabeled datasets. It can extract high-level features from the dataset as a result. The CNN model is the most common method for analyzing medical images [15]. The backpropagation calculation enables the CNN model to adaptively learn spatial highlights in clinical images. 

For ALL detection models to acquire characteristic spatial domain features, they must be trained with sufficient images. DL methods work well when many images are available for learning [16]. The use and availability of images for training are considered when selecting the hyperparameters and the model parameters. After that, the model uses the utilized dataset to learn from and classify images by updating the new weights for the specified number of classes following each training step. To improve the performance of the artificial neural network (ANN) model in specific applications, hyperparameters such as the learning rate (LR), batch size (BS), and momentum need to be fine-tuned. The most critical hyperparameter used to improve model accuracy during CNN model training is the LR. The conventional LR strategies of exponential decay, step decay, and constant LR use trial and error to determine the application-appropriate LR. Model learning with a fixed LR strategy is utilized as a baseline method rather than its alternatives. The model converges slowly when the LR is too low, and when the LR is too high, the model learning diverges, resulting in inadequate solutions. The network converges in fewer iterations at optimal LRs. The LR determines the extent of the loss gradient that is backpropagated to move to global minima. When the gradient reaches its local minimum, only the computational cost of making progress is worth it. If there is no improvement in accuracy after a few epochs or if the LR remains stuck at local minima, adaptive LR training methods use an LR that fluctuates by a predetermined value. In contrast, in the nonadaptive schedule, the LR will either decrease gradually over time at each epoch in small steps or remain constant throughout the training.

The cyclical learning rate (CLR) [17], stochastic gradient descent (SGD) with warm restarts (SGDWR) [18], also known as stochastic weight averaging (SWA), and cosine annealing are three additional dynamic LR methods that have recently emerged [19]. During training, the CLR fluctuates between predetermined upper and lower boundary values. The LR is kept at a very low level but gradually rises until it reaches its highest point. In contrast, in the nonadaptive schedule, the LR decreases gradually at each epoch in small steps or remains constant throughout the training.

The LR then drops back to the underlying worth of finishing one cycle. Consequently, a cycle has two steps and a fixed step size—the number of loops over which the LR rises to its highest value. The pattern repeats itself after each training cycle until the final epoch of the triangular LR. While increasing the LR will affect accuracy in the short term, it will also reduce training-related loss in the long run [17].

**Figure 2:**
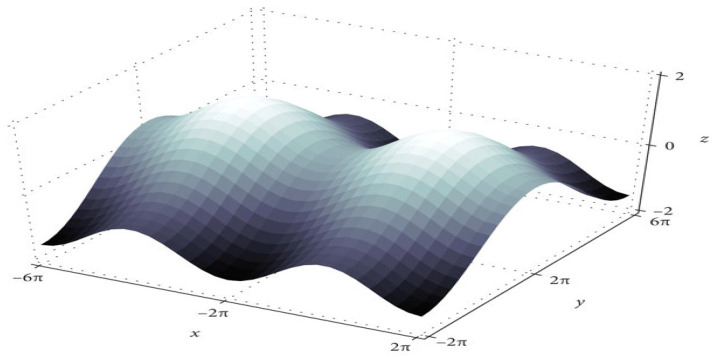
The saddle point in the loss landscape.

The training algorithm for deep neural networks (DNN) is the DNN with SGD [18]. After each epoch, the optimizer updates the parameters. Figure 2 shows that convergence time increases at saddle point plateaus when the learning rate is low, even though optimization occurs in small steps. In nonconvex optimization problems, avoiding saddle points is possible by increasing the learning rate. 

Another modality of the dynamic learning rate schedule is cosine annealing [19]. The annealing schedule depends on the cosine function and begins with a large learning rate that gradually decreases to a minimum value before rapidly increasing again. The cosine annealing schedule is represented by Equation (1). For the ith run, each batch’s learning rate decreases with cosine annealing.

η_t_ = η^i^ _min_ + 0.5 (η^i^ _max_ − η^i^ _min_)(1 + cos(*T*_cur_*π/*T_i_*) (1)

where η^i^ _min_ and η^i^ _max_ are the learning rate ranges and *T*_cur_ is how many epochs have passed since the most recent restart [20].

In this research, we set up a custom LR that compared the loss value and training accuracy at the beginning of each epoch to that of the previous epoch. We increased the LR if they were smaller and decreased the LR if they were larger. 

In this research, we proposed a fully automated solution based on the CNN model to classify ALL cell images automatically. The proposed model used the EfficientNet-B3 model to classify images of ALL and healthy cells. The C-NMC_Leukemia dataset was pre-processed with normalization and balancing to train the designed DL model from scratch to find the most relevant parameter values and improve network convergence. By manipulating the LR, we investigated the best settings for the EfficientNet-B3 model to achieve a high classification accuracy. During the training phase, we used dynamic learning. We set up a custom LR that compared the loss value and training accuracy at the beginning of each epoch to that of the previous epoch. We increased the LR if they were smaller and decreased the LR if they were larger. In addition to the detection of ALL, the proposed model was used to distinguish between uninfected and parasitized microscopic images with an average accuracy of 97.68%. As a result, the model performed better and achieved a relatively high-level performance than traditional methods with a fixed LR. The following is a summary of the contributions made in this research:

- For ALL disease prediction, a robust model using the EfficientNet-B3 CNN model and dynamic LR was proposed to distinguish between benign and malignant cells accurately and reliably.
- We compared the proposed model with five other techniques: EfficientNet-B0, EfficientNet-B1, EfficientNet-B2, InceptionResNetV2, and DenseNet121.
- With an average accuracy of 97.68%, the proposed model differentiated between parasitized and uninfected microscopic images.

The remainder of this paper is formulated as follows. Section 2 presents a literature review of CAD diagnostic systems. Section 3 displays the methodology of the proposed framework, the pre-processing of the two datasets, and the training of five classifiers. The experimental results of the proposed framework are shown in Section 4. Finally, the conclusion of our proposed framework is provided in Section 5.

## 2. Literature Review

Abir et al. [1] proposed a model to recognize ALL as an automated method that used various transfer learning models. This method employs local interpretable model-agnostic explanations (LIME) to guarantee reliability and validity. The proposed technique using the InceptionV3 model achieved an accuracy of 98.38%. Different approaches to transfer learning, such as InceptionResNetV2, VGG19, and ResNet101V2, were tested, and the results were found to be consistent with the proposed method using the LIME algorithm for explainable artificial intelligence (XAI). 

In Mondal et al. [4], the ALL-recognition task was automated using CNN models. A weighted ensemble of deep CNN models was investigated to recommend a better ALL-cell classifier. A variety of data augmentations and pre-processing was incorporated to improve the network’s generalizability. The C-NMC-2019 ALL dataset was used to train and evaluate the proposed model. The proposed model had an area under the curve (AUC) of 0.948, a balanced accuracy of 88.3%, and a weighted F1-score of 89.7%. On the other hand, the ensemble models, such as InceptionResNet-V2, DenseNet-121, Xception, MobileNet, and VGG-16, typically had coarse and scattered learned areas not present in the ensemble.

A non-invasive, medical image-based diagnosis method based on CNN models was presented by Amin et al. [12]. A CNN-based model was used in the proposed solution to extract higher-quality features from the dataset using a module called efficient channel attention (ECA) and the visual geometry group from Oxford (VGG16). The proposed approach demonstrated that the ECA module aided in overcoming the morphological similarities that exist between images of healthy cells and ALL cancer. Training data quantity and quality were also increased using various augmentation methods. The proposed CNN model successfully extracted in-depth features with an accuracy of 91.1%. The results demonstrated that pathologists would gain from the proposed method’s ability to diagnose ALL.

Khandekar et al. [21] proposed an automation system using AI to automate the detection of blast cells. A method for object detection was incorporated into the proposed automation system, using images of microscopic blood smears to predict leukemic cells. The authors used the You Only Look Once (YOLO) algorithm for cell classification and detection in its fourth version. As a result, the classification was performed as a binary problem, with each cell classified as either healthy cells (HEM) or blast cells (ALL). Images from the ALL_IDB1 and C_NMC_2019 datasets were training and testing grounds for the Object Detection algorithm. The ALL-IDB1 dataset had a mean average precision (mAP) of 96.06%, while the C_NMC_2019 dataset had an mAP of 98.7%. During pre-screening, this proposed blast cell detection algorithm could help identify leukemia from microscopic blood smear images.

Almadhor et al. [22] used naive Bayes (NB), K-nearest neighbor (KNN), random forest (RF), and support vector machine (SVM) in proposing an ensemble automated prediction strategy. They used the C-NMC leukemia dataset from Kaggle. The C-NMC leukemia dataset is broken up into two groups: healthy and cancer cells. The results showed that SVM outperforms other algorithms with an accuracy of 90%.

Kasani et al. [23] proposed an aggregated DL model to classify leukemic B-lymphoblasts. The authors used data augmentation methods to create more training samples for the small dataset. A transfer learning concept was used to accelerate the training process and further improve the proposed network’s results to create a deep learner that was both reliable and accurate. With a test dataset accuracy of 96.58% for Leukemic B-lymphoblast diagnosis, the proposed method outperformed individual networks by combining features from the best deep learning models.

Liu et al. [24] proposed a ternary stream fine-grained classification model to differentiate lymphoblasts from normal white blood cells and reactive lymphocytes. The proposed model is based on microscopic images of peripheral blood smears. Using the C-NMC dataset, the model achieved outstanding accuracy (approximately 91.90%) and showed a promising performance in distinguishing morphological cell types. 

From the previous review of the current studies conducted recently, we can conclude that no study achieved ideal classification of ALL diseases and detection of malarial parasites. However, with the C-NMC_Leukemia dataset, our proposed model achieved an average precision, recall, specificity, accuracy, and Disc similarity coefficient (DSC)98.29% or 97.83%, 97.82%, 98.31%, and 98.05%, respectively. Moreover, with the National Institutes of Health (NIH) dataset, the proposed model achieved an average precision, recall, specificity, accuracy, and DSC of 97.69%, 97.68%, 97.67%, 97.68%, and 97.68%, respectively.

## 3. Materials and Methods

### 3.1. Datasets Description

The proposed model used the C-NMC_Leukemia dataset [11] for ALL-cell prediction and the Giemsa-stained thin blood images from the dataset created by the NIH for malaria diagnosis [25]. ALL causes about 25% of pediatric cancers. Under the microscope, it is generally hard to differentiate between normal cells and immature leukemic blasts due to the similar morphological appearance of the two types of cells. An experienced oncologist assigned a normal or malignant classification to images in this dataset. The C-NMC_Leukemia dataset was divided into three sets: the training set with 9594 (90%) images, the validation set with 533 images (0.05%), and the test set with 534 images (0.05%). The NIH’s dataset images were taken from 50 healthy people and 150 people with *Plasmodium falciparum* infection. The dataset has a total of 27,558 images of red blood cells. Thirteen thousand seven hundred seventy-nine people were infected, and 13,779 were not. NIH’s dataset was divided into three sets: the training set with 24,804 (90%) images, the validation set with 1378 images (0.05%), and the test set with 1378 images (0.05%).

### 3.2. Model Architecture and Training

**Figure 3:**
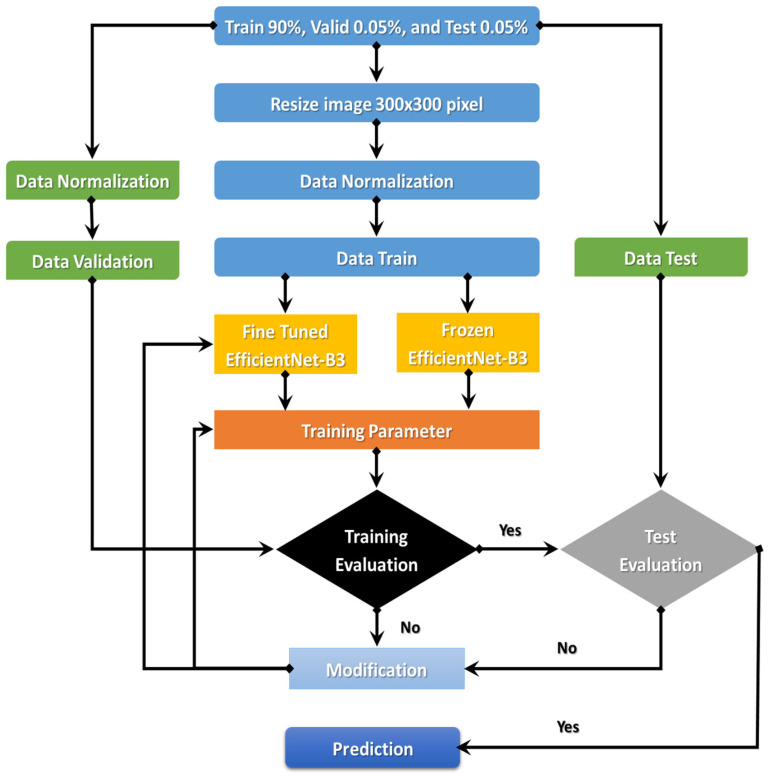
Flowchart of the proposed model.

The EfficientNet-B3 is currently demonstrating true success in classification. An automated system for processing C-NMC_Leukemia’s images to identify ALL-cell disease is the goal of the comprehensively proposed model. The following sections will discuss the proposed model. Figure 3 details the proposed model’s steps: (1) image pre-processing, (2) dataset normalization, (3) model training, (4) training evaluation, and (5) test evaluation.

The proposed model began with downloading the two image datasets and continued with image pre-processing. The two image datasets were pre-processed with normalization, contrast handling, resizing, and artifact removal. Pre-processing the two datasets effectively yields accurate results, making it one of the most important steps. After pre-processing the two datasets, we divided them into the training set (90%), validation set (0.05%), and test set (0.05%). In the third step, we used transfer learning to train the EfficientNet- B3. The first step of transfer learning is supervised pre-training, in which we downloaded the ImageNet neural network with parameters that had been trained beforehand on a large dataset. In the second step of the transfer learning, we used the C-NMC_Leukemia and the NIH datasets to fine-tune the EfficientNet-B3 network. In the fourth step, we calculated the training dataset’s error. If the training dataset’s error was not low, we re-trained the model. If the training dataset’s error was low, we calculated the test dataset’s error. If the test dataset’s error was not low, we re-trained the model. Table 1 lists the algorithm for the dynamic LR. 

#### 3.2.1. Data Pre-Processing

In this research, data pre-processing was necessary since the images differ in resolution, pixel-level noise, size, bright text, and symbols. An image mask was executed on the images to address such artifacts using Equation (2). Moreover, the contrast of images might differ. The training images’ contrasts were normalized in the training phase to solve this problem. After that, we filtered the images to remove noise. Each pixel image was precisely subtracted from the three main colors’ average: red, green, and blue (RGB).

(2) $$Mask\left(m,n\right)=\{\begin{array}{c} \max_{i}\;,i\left(m,n\right)\geq \min_{i}\;\\ 0\;otherwise. \end{array}$$

**Figure 4:**
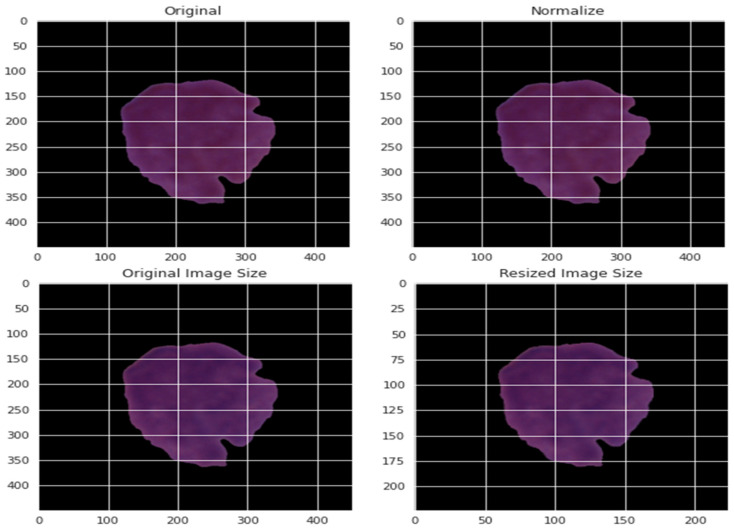
An example of applying the pre-processing stage on blood cells.

By normalizing the data in various ways, the effect of various pixel intensities can be defined. The normalized data PI* is obtained using the normalization method from the pixel intensity recorded as PI. This pixel has a value that ranges from 0 to 255 for each of the three primary colors. Consequently, the value of each pixel was divided by 255 to achieve normalization. The normalization was based on the maximum and minimum values of the experiment, as presented by Equation (3). The pictures were resized to a decent goal of 300 by 300. Figure 4 depicts a blood cell before and after the pre-processing.

(3) $$P_{I}^{*}=\left(P_{I}{}_{l}-Min_{old}\right)\frac{Max_{new}-Min_{new}}{Max_{old}-Min_{old}}+Min_{new},\;l\in \left[0,n\right]$$

#### 3.2.2. EfficientNet-B3

Recently, Tan and Le [26] investigated the connection between the width and depth of CNN models and devised an effective approach for designing CNN models with fewer parameters but a greater classification accuracy. They proposed seven such models, which they referred to as EfficientNet-B0 to EfficientNet-B7. They referred to them as EfficientNet CNN models. When EfficientNet CNN models were applied to the ImageNet dataset, they demonstrated that their models outperformed all recent models regarding the number of Top-1 accuracy and parameters [27]. 

A novel approach to scaling CNN models is the foundation for the EfficientNet family. It makes use of a straightforward yet powerful compound coefficient. Uniquely in contrast to conventional strategies that scale aspects of organizations, such as width, profundity, and goal, EfficientNet scales each aspect with a proper set of scaling coefficients consistently. Scaling individual aspects works on model execution but adjusting all organization components regarding the accessible assets works on overall execution.

EfficientNet is much smaller than other models, with ImageNet accuracy comparable to its own. For instance, the ResNet50 model, as found in the Keras application, has 23,534,592 boundaries. Still, it needs to meet the expectations of the littlest EfficientNet (called EffecientNet-B0), which has 5,330,564 boundaries. We proposed an effective model based on the EfficientNet-B3 CNN model because it strikes a good balance between accuracy and computational power [27].

The primary component of the EfficientNet model family is mobile inverted bottleneck convolution (MBConv). The MobileNet models’ concepts were the foundation for MBConv [28]. One central idea was to use depthwise separable convolutions, which entailed layering a pointwise and a depthwise convolution. The following two additional concepts were taken from MobileNet-V2, the second improved version of MobileNet: (1) residual connections that were inverted and (2) linear bottlenecks.

The EfficientNet model family begins with its stem, which is where all the experimenting with the architecture starts. The stem is common to all eight models and the final layers. 

**Figure 5:**
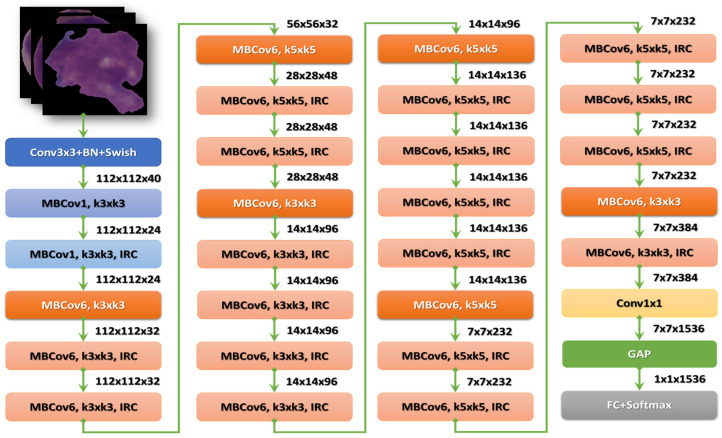
The structure of the EfficientNet-B3 model.

After the stem, there are seven blocks. Additionally, these blocks have varying sub-blocks, and their number grows as they progress from EfficientNet-B0 to EfficientNet-B7. The total number of layers in EfficientNet-B0 is 237, while the total number in EfficientNet-B7 is 813. The second module is the foundation for the first sub-block of the seven main blocks, except the first. Module 3 is connected to all the sub-blocks via a skip connection. The skip connection in the first sub-blocks is combined with this Module 4. Module 5 brings together each sub-block by connecting it in a skip fashion to the one before it. Finally, sub-blocks are created by combining these modules with being used in particular ways in the blocks [27]. Figure 5 shows the structure of the EfficientNetB3 model.

#### 3.2.3. Dwell

When the validation loss on the current epoch is greater than that on the previous epoch, the DWELL callback can be useful for training a model. The proposed model has reached a point in N space (where N is the number of trainable parameters) that is less favorable than that for the previous epoch when that occurs. The callback checks for this condition and sets the model weights to those of the epoch with the least amount of validation loss if it finds it. Additionally, it slows down learning. We will remain at the same unfavorable point in N space if the LR for the subsequent epoch is kept.

## 4. Model Implementation and Evaluation 

### 4.1. Model Evaluation Metrics

The proposed model was evaluated using the accuracy, precision, sensitivity, specificity, and DSC, which are presented in Equations (4)–(8).

**Accuracy** = (TP + TN)/(TP + TN + FP + FN) (4)

Precision = TP/(TP + FP)(5)

Sensitivity = TP/(TP + FN)(6)

Specificity = TN/(TN + FP)(7)

DSC = (2 TP)/(2 ∗ TP + FP + FN)(8)

True positive, true negative, false positive, and false negative are represented by TP, TN, FP, and FN, respectively. Precision is defined as how much of the samples correctly predicted by the model would be positive. Sensitivity is defined as the ratio of the number of actual positives to the number of true positives. Specificity is defined as the ratio of the number of actual negatives to the number of true negatives. DSC is the harmonic average of recall and precision.

### 4.2. Model Implementation

The C-NMC_Leukemia dataset was split into 70% for the training, 17% for the testing, and 12% for the validation. The implementation was carried out in the Kaggle environment. The characteristics of the PC used for the experiments are x64-based Intel (R) Core (TM) i7-10510U CPU with 1.80 GHz and 2.30 GHz, 16 GB of memory, and a 64-bit Windows platform.

Table 2 shows the experiments’ results for EfficientNet-B3 and five other CNN models: EfficientNet-B0, EfficientNet-B1, EfficientNet-B2, InceptionResnetV2, and DenseNet121 using fixed LR. All CNN models were applied to the C-NMC_Leukemia dataset for the binary classification, in which we distinguished benign and malignant cells. Table 2 shows that EfficientNet-B3, EfficientNet-B0, EfficientNet-B1, EfficientNet-B2, InceptionResnetV2, and DenseNet121 had accuracy averages of 97.57%, 93.82%, 94.38%, 95.51%, 93.07%, and 82.21%, respectively. The EfficientNet-B3 model had the highest precision, recall, accuracy, and DSC average, equal to 97.42%, 96.96%, 97.57%, and 97.18%, respectively. The EfficientNet-B2 model had the highest average specificity, equal to 98.90%.

Table 2 shows the binary classification results of the six CNN models, EfficientNet-B3, EfficientNet-B0, EfficientNet-B1, EfficientNet-B2, InceptionResnetV2, and DenseNet121, on the C-NMC_Leukemia dataset. This experiment distinguished ALL (cancer) and Hem (healthy) classes.

For the ALL class, precision, recall, specificity, accuracy, and DSC were 97.82%, 98.63%, 95.29%, 97.57%, and 98.22% for EfficientNet-B3, respectively. EfficientNet-B3 and EfficientNet-B1 had the highest precision, 97.01% and 99.28%, respectively. EfficientNet-B3, EfficientNet-B0, and EfficientNet-B2 had the highest recall of 98.63%, 99.73%, and 98.9%, respectively. EfficientNet-B3 and EfficientNet-B1 achieved the highest specificity, 95.29% and 95.8%, respectively. EfficientNet-B3 and EfficientNet-B2 had the highest accuracy, 97.57% and 95.5%, respectively. EfficientNet-B3 and EfficientNet-B2 achieved the highest DSC, equal to 98.22% and 96.77%, respectively. 

**Figure 6:**
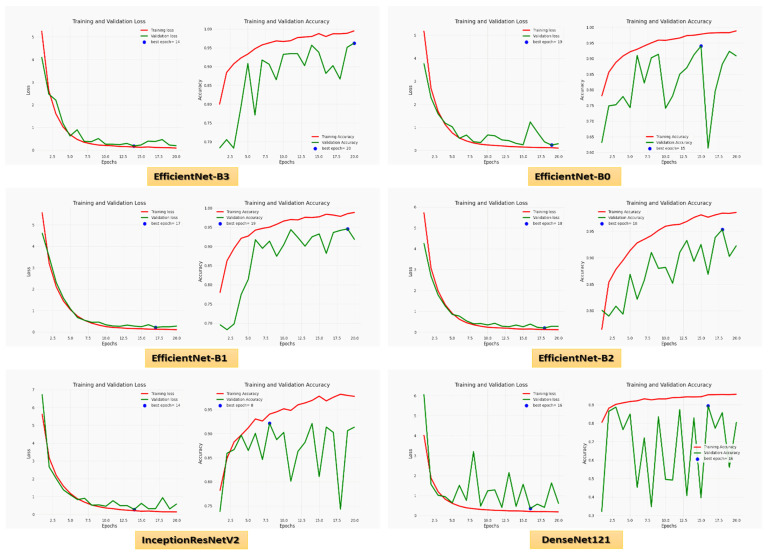
The loss and accuracy curves for the six CNN models using a fixed LR.

For the Hem class, precision, recall, specificity, accuracy, and DSC were 97.01%, 95.29%, 98.63%, 97.57%, and 96.14% for EfficientNet-B3, respectively. EfficientNet-B3 and EfficientNet-B0 had the highest precision, 97.82% and 97.99%, respectively. EfficientNet-B3, EfficientNet-B1, and EfficientNet-B2 had the highest recall of 95.29%, 95.88%, and 95.51%, respectively. EfficientNet-B3, EfficientNet-B0, EfficientNet-B2, and InceptionResNetV2 achieved the highest specificity of 98.63%, 99.73%, 98.90%, and 97.53%, respectively. EfficientNet-B3 and EfficientNet-B2 had the highest accuracy, 97.57% and 95.51%, respectively. EfficientNet-B3 and EfficientNet-B2 achieved the highest DSC, 96.14% and 92.59%, respectively. Figure 6 shows the training and validation losses and accuracy of the six CNN models using a fixed LR.

**Figure 7:**
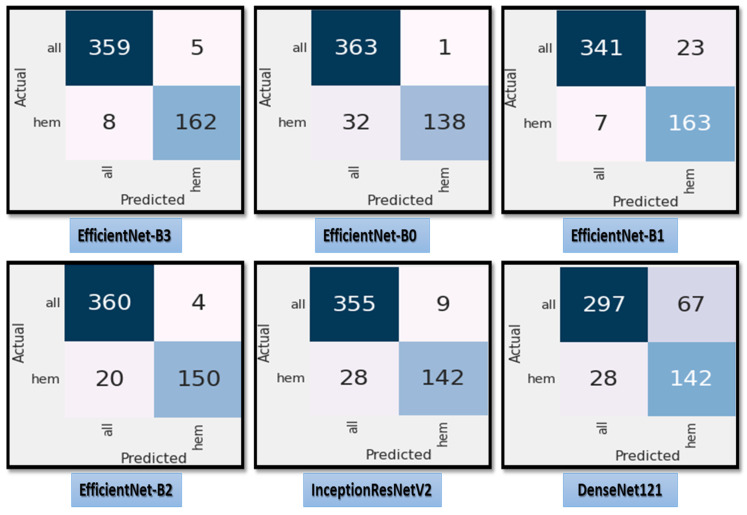
**The** test dataset’s confusion matrix for the six CNN models using a fixed LR.

Figure 7 shows the test dataset’s confusion matrix for the six CNN models using the fixed LR. The test dataset was classified into two classes: ALL (cancer) with 364 images and Hem (healthy) with 170 images. For class ALL, the accuracy of the EfficientNet-B3 model was 98.6%, as it predicted 359 images correctly out of 364. The accuracy of the EfficientNet-B0 model was 99.7%, as it predicted 363 images correctly. The accuracy of the EfficientNet-B1model was 93.6%, as it predicted 341 images correctly. The accuracy of the EfficientNet-B2 model was 98.9%, as it predicted 360 images correctly. The accuracy of the InceptionResNetV2 model was 97.5%, as it predicted 355 images correctly, and the accuracy of the DenesNet121 was 81.5%, as it predicted 297 images correctly.

Table 3 depicts the outcomes of the experiments for EfficientNet-B3 and five additional CNN models using a dynamic LR, EfficientNet-B0, EfficientNet-B1, EfficientNet-B2, InceptionResnetV2, and DenseNet121. We used all CNN models on the C-NMC_Leukemia dataset for the binary classification to distinguish between benign and malignant cells. 

Table 3 shows that EfficientNet-B3, EfficientNet-B0, EfficientNet-B1, EfficientNet-B2, InceptionResnetV2, and DenseNet121 had average accuracies of 98.31%, 97.57%, 97.19%, 97.00%, 95.32%, and 94.38%, respectively. The EfficientNet-B3 model had the highest average of precision, recall, accuracy, and DSC at 98.29%, 97.83%, 98.31%, and 98.05%, respectively. EfficientNet-B1 and EfficientNet-B2 achieved the highest average specificity, 98.08%. In Table 3, we distinguished between two classes in this experiment: Hem (healthy) and ALL (cancer). In Table 4, we compared in accuracy among the six CNN models using the proposed dynamic LR and using the fixed LR for ALL diseases.

For the ALL class, precision, recall, specificity, accuracy, and DSC were 98.37%, 99.18%, 96.47%, 98.31%, and 98.77% for EfficientNet-B3, respectively. The precision of EfficientNet-B3, EfficientNet-B0, EfficientNet-B1, and EfficientNet-B2 was 98.37%, 98.37%, 97.81%, and 97.54%, respectively. EfficientNet-B3, EfficientNet-B0, and InceptionResNetV2 had the highest recall, with 99.18%, 99.18%, and 98.35%, respectively. EfficientNet-B3 and EfficientNet-B0 achieved specificity scores of 96.47%. The most accurate models, EfficientNet-B3, EfficientNet-B0, and EfficientNet-B1, were 98.31%, 98.31%, and 97.19%, respectively. EfficientNet-B3, EfficientNet-B0, and EfficientNet-B1 accomplished the most elevated DSC, with 98.77%, 98.77%, and 97.94%, respectively. 

For the Hem class, precision, recall, specificity, accuracy, and DSC were 98.20%, 96.47%, 99.18%, 98.31%, and 97.33% for EfficientNet-B3, respectively. EfficientNet-B3 and EfficientNet-B0 achieved the highest precision, recall, accuracy, and DSC, at 98.20%, 96.47, 98.31%, and 97.33%, respectively. The most specific results were obtained by EfficientNet-B3, EfficientNet-B0, and InceptionResNetV2, with 99.18%, 99.18%, and 98.35%, respectively.

**Figure 8:**
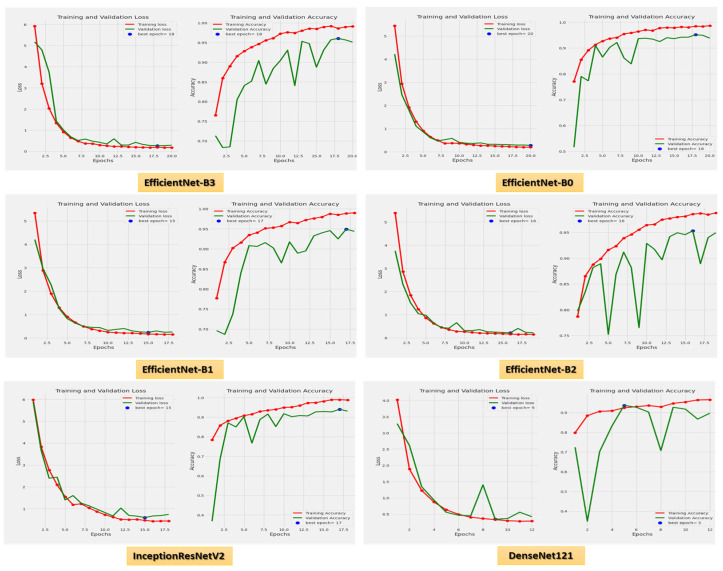
The loss and accuracy curves for the six CNN models using dynamic LR.

**Figure 9:**
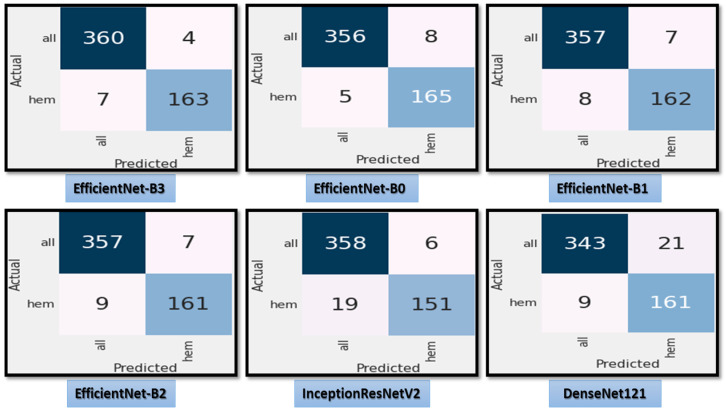
The test dataset’s confusion matrix for the six CNN models using dynamic LR.

Figure 8 demonstrates the accuracy and loss for the six CNN models trained and validated at a dynamic LR. Figure 9 shows the confusion matrix for the six CNN models that used a dynamic LR in the test dataset. There were two classes of the test dataset: 364 images in the ALL (cancer) class and 170 in the Hem (healthy) class. The EfficientNet-B3 model correctly predicted 360 images out of 364 for class ALL, with an accuracy of 98.9%. The EfficientNet-B0 model correctly predicted 356 images, giving it an accuracy of 97.8%. The EfficientNet-B1 and the EfficientNet-B2 models correctly predicted 357 images, achieving an accuracy of 98%. The InceptionResNetV2 model was 98.3% accurate, correctly predicting 358 images, while the DenesNet121 model was 94.2% accurate, correctly predicting 343 images.

After we experimented with the proposed classification model based on the EfficientNet-B3 CNN to predict ALL diseases, we experimented with the proposed model to classify uninfected and parasitized microscopic images on Giemsa-stained thin blood images from the dataset created by the NIH model. The outcomes of the experiment for EfficientNet-B3 and the five CNN models (EfficientNet-B0, EfficientNet-B1, EfficientNet-B2, InceptionResnetV2, and DenseNet121) using a fixed LR are presented in Table 5. EfficientNet-B3, EfficientNet-B0, EfficientNet-B1, EfficientNet-B2, InceptionResnetV2, and DenseNet121 achieved averaged accuracies of 85.22%, 92.91%, 96.60%, 75.89%, 75.89%, 97%, 97.19% respectively. The precision, recall, specificity, accuracy, and DSC average of the EfficientNet-B3 model were 88.70%, 85.22%, 85.11%, 85.22%, and 85.49%, respectively. The models with the highest average precision were Efficient-Net-B1 and Dense-Net121 at 97.18%. The model with the highest average recall was EfficientNet-B2 at 99.57%. The models with the highest average specificity were EfficientNet-B1 and Dense-Net121 at 97.17%. Dense-Net121 achieved the highest average accuracy and DSC with 97.19% and 97.17%, respectively. 

Table 6 portrays the results of the test dataset for EfficientNet-B3 and five other CNN models utilizing a dynamic LR. We performed the binary classification using all CNN models to differentiate between uninfected and parasitized microscopic images on Giemsa-stained thin blood images from the dataset created by the NIH. EfficientNet-B3, EfficientNet-B0, EfficientNet-B1, EfficientNet-B2, InceptionResnetV2, and DenseNet121 achieved averaged accuracies of 97.68%, 97.61%, 97.31%, 97.39%, 97.10%, 97.61% respectively. The precision, recall, specificity, accuracy, and DSC average of the EfficientNet-B3 model were 97.69%, 97.68%, 97.67%, 97.68%, and 97.68%, respectively. EfficientNet-B3 achieved the highest average recall, specificity, accuracy, and DSC, while EfficientNet-B2 achieved the highest precision. In Table 7, we presented a comparison of accuracy of the six CNN models using the proposed dynamic LR and using the fixed LR for malaria parasite 

### 4.3. Model Result Comparison with the Literature

Regarding thte binary classification accuracy, Atefeh et al. [29] used a system that recommends diagnoses automatically with an accuracy of 93.12%. Efthakhar et al. [30] used a machine-learning-based statistical model from a patient’s genome series with an accuracy of 95%. Therefore, the proposed EfficientNet-B3 model outperformed the most recently listed methods, as shown in Table 8. It had a remarkable accuracy rate of 98.31% for ALL predictions and 97.68% for malaria detection. Moreover, from the patient’s point of view, our model would improve the procedure by reducing the cost of diagnosis, speeding up diagnosis, and delaying the progression of the disease. The evaluation of the proposed model revealed that it is an unparalleled perceptive result with tuning compared to other ongoing models. Moreover, we compared the proposed model and different models that used dynamic LR in Table 8

## 5. Conclusions

This paper proposed a robust classifier to distinguish ALL as an automated model that changes the LR automatically. The classification model is based on the EfficientNet-B3 CNN model. A custom LR that compared the loss value at the beginning of each epoch to that of the previous epoch was set up in the proposed model. If it was smaller, we increased the LR; if it was larger, we decreased it. Consequently, the model performed better than a conventional method with a fixed LR and achieved a relatively high level of performance. The proposed model was evaluated using the C-NMC_Leukemia dataset, and we used normalization and balancing to pre-process the dataset. Recent classifiers were evaluated and compared to the proposed model. Our proposed model’s precision, recall, specificity, accuracy, and DSC were 98.29%, 97.83%, 97.82%, 98.31%, and 98.05%, respectively. The proposed model will help clinicians use the knowledge they extract from data stores to aid in the accurate and efficient diagnosis of ALL.. Moreover, the proposed model was used to examine microscopic images of the blood to identify the malaria parasite. Our proposed model’s average precision, recall, specificity, accuracy, and DSC were 97.69%, 97.68%, 97.67%, 97.68%, and 97.68%, respectively. The prediction time is a limitation of our proposed framework. Therefore, our future work is to improve the prediction time of the proposed framework by utilizing more optimization and feature selection approaches. Furthermore, we will use a moving average loss value by aggregating loss values across the previous N epochs.

## Figures and Tables

**Table 1:** The dynamic LR algorithm.

The Dynamic Learning Rate Algorithm
Input: no_epoch, factor (factor between 0.0 and 1.0.) Output: Dynamically Learning Rate. Begin: 1-**while** (no_epoch > 0) 1.1-**while** (no_epoch == 1) 1.1.1- Input: I (H to halt training or N to continue training). 1.1.2 - **If** (I == N) 1.1.2.1-no _epoch = N. 1.1.3- **else** 1.1.3.1- Exit **End if** **End while** 2- Input: dwell 3- **if** dwell == True 3.1 calculate curr_valid_loss 3.2 calculate curr_train_accu 3.3- **if** (curr_valid_loss > prev_valid_loss) 3.3.1- curr_W = prev_W 3.3.2- curr_B = prev_B 3.3.3- next_lr = current_lr * factor ** End if** 3.4- **if** (curr_train_accu < prev_train_accu) 3.4.1-curr_W = prev_W 3.4.2-curr_B = prev_B 3.4.3- next_lr = current_lr * factor ** End if** **End if** 4- no_epoch- = 1 **End while** End.

**Table 2:** The performance of the six CNN models using a fixed LR.

Model	Class	Precision (%)	Recall (%)	Specificity (%)	Accuracy (%)	DSC (%)
**EfficientNet-B3**	all	97.82	98.63	95.29	97.57	98.22
	hem	97.01	95.29	98.63	97.57	96.14
**Average**		**97.42**	**96.96**	**96.96**	**97.57**	**97.18**
**EfficientNet-B0**	all	91.90	99.73	81.18	93.82	95.65
	hem	99.28	81.18	99.73	93.82	89.32
**Average**		**95.59**	**90.46**	**90.45**	**93.82**	**89.32**
**EfficientNet-B1**	all	97.99	93.68	95.88	9438	95.78
	hem	87.63	95.88	93.68	94.38	91.57
**Average**		**87.63**	**95.88**	**93.68**	**94.38**	**91.57**
**EfficientNet-B2**	all	94.74	98.90	88.24	95.51	96.77
	hem	95.59	95.51	98.90	95.51	92.59
**Average**		**95.59**	**95.51**	**98.90**	**95.51**	**92.59**
**InceptionResNetV2**	all	93.69	97.53	83.53	93.07	95.05
	hem	94.04	83.53	97.53	93.07	88.47
**Average**		**93.87**	**90.53**	**90.53**	**93.07**	**91.76**
**DenseNet121**	all	91.38	81.59	83.53	82.21	86.21
	hem	67.94	83.53	81.59	82.21	74.93
**Average**		**79.66**	**82.56**	**82.56**	**82.21**	**80.57**

**Table 3:** Performance of the six CNN models using the proposed dynamic LR.

Model	Class	Precision (%)	Recall (%)	Specificity (%)	Accuracy (%)	DSC (%)
**EfficientNet-B3**	all	98.37	99.18	96.47	98.31	98.77
	hem	98.20	96.47	99.18	98.31	97.33
**Average**		**98.29**	**97.83**	**97.82**	**98.31**	**98.05**
**EfficientNet-B0**	all	98.37	99.18	96.47	98.31	98.77
	hem	98.20	96.47	99.18	98.31	97.33
**Average**		**95.38**	**97.06**	**97.80**	**97.57**	**96.21**
**EfficientNet-B1**	all	97.81	98.08	95.29	97.19	97.94
	hem	95.86	95.29	98.08	97.19	95.58
**Average**		**95.86**	**95.29**	**98.08**	**97.19**	**95.58**
**EfficientNet-B2**	all	97.54	98.08	94.70	97.00	97.80
	hem	95.83	94.71	98.08	97.00	95.27
**Average**		**95.83**	**94.71**	**98.08**	**97.00**	**95.27**
**InceptionResNetV2**	all	94.96	98.35	88.82	95.32	96.63
	hem	96.18	88.82	98.35	95.32	92.35
**Average**		**95.57**	**93.59**	**93.59**	**95.32**	**94.49**
**DenseNet121**	all	97.44	94.23	94.71	94.38	95.81
	hem	88.46	94.71	94.23	94.38	91.48
**Average**		**92.95**	**94.47**	**94.47**	**94.38**	**93.64**

**Table 4:** Comparison of accuracy of the six CNN models using the proposed dynamic LR and using the fixed LR for ALL diseases.

Model	Class	Accuracy (%) of Fixed LR	Accuracy (%) of Dynamic LR
**EfficientNet-B3**	all	97.57	98.31
	hem	97.57	98.31
**Average**		**97.57**	**98.31**
**EfficientNet-B0**	all	93.82	98.31
	hem	93.82	98.31
**Average**		**93.82**	**97.57**
**EfficientNet-B1**	all	9438	97.19
	hem	94.38	97.19
**Average**		**94.38**	**97.19**
**EfficientNet-B2**	all	95.51	97.00
	hem	95.51	97.00
**Average**		**95.51**	**97.00**
**InceptionResNetV2**	all	93.07	95.32
	hem	93.07	95.32
**Average**		**93.07**	**95.32**
**DenseNet121**	all	82.21	94.38
	hem	82.21	94.38
**Average**		**82.21**	**94.38**

**Table 5:** The performance of binary classification of malaria parasite for the six CNN models using a fixed LR.

Model	Class	Precision (%)	Recall (%)	Specificity (%)	Accuracy (%)	DSC (%)
**EfficientNet-B3**	Parasitized	99.39	70.86	82.73	99.57	85.49
	Uninfected	78.01	99.57	87.48	70.86	85.49
**Average**		**88.70**	**85.22**	**85.11**	**85.22**	**85.49**
**EfficientNet-B0**	Parasitized	99.15	86.54	92.42	99.29	93.03
	Uninfected	88.45	99.29	93.56	86.54	93.03
**Average**		**93.80**	**92.92**	**92.99**	**92.91**	**93.03**
**EfficientNet-B1**	Parasitized	97.61	96.6	97.1	97.72	97.16
	Uninfected	96.76	97.72	97.24	96.60	97.17
**Average**		**97.18**	**97.16**	**97.17**	**97.16**	**97.16**
**EfficientNet-B2**	Parasitized	99.42	75.89	86.07	99.57	87.95
	Uninfected	81.09	99.57	89.39	75.89	87.95
**Average**		81.09	99.57	89.39	75.89	87.95
**InceptionResNetV2**	Parasitized	**98.03**	**95.86**	**96.93**	**98.14**	**97.02**
	Uninfected	96.09	98.15	97.11	95.86	97.02
**Average**		**97.06**	**97.01**	**97.02**	**97.00**	**97.02**
**DenseNet121**	Parasitized	95.96	98.37	97.15	96.01	97.17
	Uninfected	98.39	96.01	97.19	98.37	97.17
**Average**		**97.18**	**97.19**	**97.17**	**97.19**	**97.17**

**Table 6:** The performance of binary classification of malaria parasites for the six CNN models using the proposed dynamic LR.

Model	Class	Precision (%)	Recall (%)	Specificity (%)	Accuracy (%)	DSC (%)
**EfficientNet-B3**	Parasitized	97.92	97.34	98.01	97.67	97.63
	Uninfected	97.45	98.01	97.34	97.68	97.73
**Average**		**97.69**	**97.68**	**97.67**	**97.68**	**97.68**
**EfficientNet-B0**	Parasitized	98.35	96.75	98.43	97.61	97.54
	Uninfected	96.91	98.43	96.74	97.61	97.67
**Average**		**97.63**	**97.59**	**97.59**	**97.61**	**97.67**
**EfficientNet-B1**	Parasitized	97.33	97.19	97.43	97.31	97.26
	Uninfected	97.30	9744	97.19	97.31	97.37
**Average**		**97.30**	**97.44**	**97.19**	**97.31**	**97.37**
**EfficientNet-B2**	Parasitized	97.06	97.63	97.15	97.38	97.34
	Uninfected	97.71	97.15	97.63	97.39	97.43
**Average**		**97.71**	**97.15**	**97.63**	**97.39**	**97.43**
**InceptionResNetV2**	Parasitized	97.89	96.15	98.01	97.09	97.01
	Uninfected	96.36	98.01	96.15	97.10	97.18
**Average**		**97.13**	**97.08**	**97.08**	**97.10**	**97.10**
**DenseNet121**	Parasitized	98.20	96.89	98.29	97.61	97.54
	Uninfected	97.05	98.29	96.89	97.61	97.66
**Average**		**97.63**	**97.59**	**97.59**	**97.61**	**97.60**

**Table 7:** Comparison of accuracy of the six CNN models using the proposed dynamic LR and using the fixed LR for malaria parasite.

Model	Class	Accuracy (%) of the Dynamic LR	Accuracy (%) of the Fixed LR
**EfficientNet-B3**	Parasitized	97.67	99.57
	Uninfected	97.68	70.86
**Average**		**97.68**	85.22
**EfficientNet-B0**	Parasitized	97.61	99.29
	Uninfected	97.61	86.54
**Average**		**97.61**	92.91
**EfficientNet-B1**	Parasitized	97.31	97.72
	Uninfected	97.31	96.60
**Average**		**97.31**	97.16
**EfficientNet-B2**	Parasitized	97.38	99.57
	Uninfected	97.39	75.89
**Average**		**97.39**	75.89
**InceptionResNetV2**	Parasitized	97.09	98.14
	Uninfected	97.10	95.86
**Average**		**97.10**	97.00
**DenseNet121**	Parasitized	97.61	96.01
	Uninfected	97.61	98.37
**Average**		**97.61**	97.19

**Table 8:** The comparison results between the proposed model and other recent models.

Study	Methodology	Tested Metrics	Datasets
Abir et al. [1]	InceptionV3	Accuracy 80%, F-Score 79.8	C-NMC _Leukemia
Mondal et al. [4]	Ensemble of Xception, VGG-16, DenseNet-121, MobileNet, and InceptionResNet-V2	88.3%	C-NMC _Leukemia
Amin et al. [12]	ECA-Net Based on VGG16	91.1%	C-NMC _Leukemia
Khandekar et al. [21]	YOLOv4, VGG16, ResNet-50, Darknet52, CSPDarknet53 or ResNext50.	For C-NMC_Leukemia dataset: Weighted F1-score on the test set of 92% with Mean Average Precision of 98.57% and recall of 96%. For ALL-IDB1 dataset: Mean Average Precision of 95.57%, Recall of 92% and F1 score of 0.92	C-NMC _Leukemia and ALL-IDB1
Almadhor et al. [22]	NB, KNN, RF, and SVM in proposing an ensemble automated prediction strategy	SVM outperforms other algorithms with an accuracy of 90%.	C-NMC _Leukemia
Liu et al. [24]	AlexNet, VGGNet, NASNet, Xception, DenseNet, InceptionV3, MobileNet, and ShuffleNet	96.58%	C-NMC _Leukemia
Tan and Le [26]	ternary stream fine-grained model	91.9%	C-NMC _Leukemia
Atefeh et al. [29]	A recommender system (MDSS)	93.12%	Data collected from Iran Blood Transfusion Organization (IBTO)
Efthakhar et al. [30]	Naive Bayes	95%	NCBI GEO dataset
proposed model	EfficientNet-B3 model	98.31%	C-NMC _Leukemia
proposed model for malaria detection	EfficientNet-B3 model	97.68%	NIH

## Data Availability

https://www.kaggle.com/datasets/avk256/cnmc-leukemia (accessed on 10 January 2023).
